# Supplementary figures and images for: Contrasting the effects of adaptation and synaptic filtering on the timescales of dynamics in recurrent networks
Source: PLoS Comput Biol. 2019 Mar 21;15(3):e1006893. doi: 10.1371/journal.pcbi.1006893 (PMC6445477; doi:10.1371/journal.pcbi.1006893)

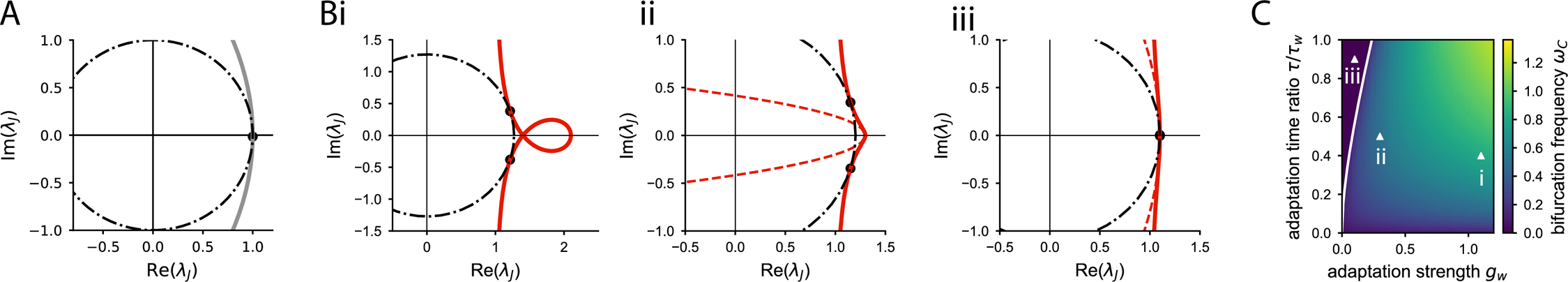

Supplement: S1 Fig — A: Instability bound for the system with synaptic filtering (grey line, Eq 72) and eigenspectrum for the weakest unstable synaptic coupling J. For any parameter combination, the instability bound, a parabola, is first touched by the growing circle of eigenvalues at ω = 1 and value JCE+g2CI=1. B: Three different configurations of the instability bound for the system with adaptation in the complex plane of eigenvalues of the connectivity matrix, λJ. The black dots indicate the intersection between the instability boundary (full red line) and the eigenspectrum of λJ (dashed black line) with weakest coupling that is unstable. (i) The instability boundary intersects the real axis twice, leading to a Hopf bifurcation. (ii) It intersects the real axis just once and still leads to a Hopf bifurcation, because the intersection with the real axis is not the closest point of the curve to the origin. (iii) It intersects the real axis once and leads to a zero-frequency bifurcation, because the crossing of the real axis is the closest point to the origin. In (ii) and (iii) we draw the parabolic approximation of the instability bound (red dashed line, Eq 82). If the curvature of this parabola is exterior to the λJ eigenspectrum, as in (iii), the system undergoes a zero-frequency bifurcation. C: Oscillatory frequency at which the network with adaptation undergoes a bifurcation. To the right of the white line (Eq 84), the network displays a Hopf bifurcation, whereas to the left, the bifurcation happens at zero-frequency. The triangles indicate the parameter combinations used in B. (TIF) [file pcbi.1006893.s001.tif]
